# Prediction of Necroptosis-Related Markers in Head and Neck Carcinoma by Bioinformatics

**DOI:** 10.1155/2022/1993023

**Published:** 2022-06-22

**Authors:** Lin Zheng, Minhui Li, Ruiyuan Gu, Haoxiang Zhang, Xiaotong Qi, Honglin Dong

**Affiliations:** ^1^Department of Vascular Surgery, The Second Hospital of Shanxi Medical University, Taiyuan 030000, China; ^2^Department of Otolaryngology Head and Neck Surgery, Shanxi Medical University Second Affiliated Hospital, Taiyuan 030000, China

## Abstract

Necroptosis is a form of programmed cell death that has recently been shown to be important in the progression of head and neck cancer (HNC). Noncoding RNAs (ncRNAs) are known to function in cell death and tumor formation. In this study, we focused on microRNAs (miRNA) that play roles in necroptosis and the progression of HNC. We collected miRNA expression data, related clinical data of patients with HNC, and miRNA data related to necroptosis. A prognostic multimiRNA molecular marker was generated based on differential expression analysis and univariate and multivariate Cox regression analyses. Target genes of the prognosis-related miRNAs were identified, and their functions were evaluated by Gene Ontology Enrichment Analysis to reveal the processes the miRNAs may be involved in. Eight potentially prognostic miRNAs were identified through differential expression analysis: miR-331-3p, miR-181d-5p, miR-181b-5p, miR-500a-3p, miR-425-5p, miR-181a-5p, miR-141-3p, and miR-200a-5p. Multivariate Cox regression identified the risk score as an independent prognostic factor (univariate Cox regression results: hazard ratio (HR): 2.2028, 95% confidence interval (CI): 1.2640–3.8388, *P* = 0.0053; multivariate Cox regression results: HR: 2.4168, 95% CI: 1.3743–4.2501, *P* = 0.0022). Survival curve analysis revealed that patients with a high risk score had a bad prognosis (*P* = 0.0109). A receiver operating characteristic curve showed that the model has a certain prediction ability. We identified 187 miRNA-related genes, which were enriched in “cell cycle” and “cellular senescence.” In conclusion, this study identified eight novel miRNA markers for predicting the prognosis of patients with HNC and paved the way for future research on necroptosis-related genes.

## 1. Introduction

Head and neck cancer (HNC) is one of the top 10 most prevalent cancers globally [1]. All HNCs originate in the upper aerodigestive tract, which includes the upper pharynx, larynx, esophagus, and thyroid as well as the corresponding lymph nodes, soft tissues, and bone [2]. More than 90% of all malignancies in the HNC category originate from squamous cell carcinomas (SCCs) and their variations. Adenocarcinomas, sarcomas, anaplastic carcinomas, plasmacytomas, lymphomas, and malignant melanomas are all histopathologically classified as other head and neck tumors. Heavy cigarette and alcohol use are responsible for approximately 75% of all cases of HNC globally [3]. HNC carcinogenesis, tumor development, and metastasis are likely influenced by a number of unknown variables. Therefore, no accurately and reliably cure is available for HNC sufferers today.

The term necroptosis was coined when small-molecule inhibitors of this death program, termed “necrostatins,” were identified [4]. Necrostatins were found to inhibit RIPK1 [5], which was consistent with earlier findings highlighting the importance of RIPK1 [6]. Necrostatin-1 (Nec-1) ameliorated both ischemic brain injury and myocardial ischemia-reperfusion injury in mice [7], suggesting a link between necroptosis and noninfectious diseases.

Programmed cell death (PCD) is a form of cell-autonomous organized death. It includes apoptosis, autophagy, necroptosis, ferroptosis, and pyroptosis [8]. It is widely accepted that apoptosis is the most common form of cell death. Necroptosis is being investigated as a possible cancer therapy target because of its important role in cancer development. An increasing number of drugs that induce necroptosis specifically in cancer cells are being developed to combat cancer. One of the most important regulators is kinase receptor-interacting protein (RIP) kinases, including RIP1 and RIP3. Nec-1 may also specifically prevent necroptosis [9].

Recent studies have shown that noncoding RNAs (ncRNAs) are involved in a variety of mechanisms that contribute to tumor metastasis, including cell cycle progression and angiogenesis [10]. NcRNAs play roles in tumor formation and metastasis by regulating the gene expression in several ways. In addition, microRNAs (miRNAs) have functions in regulating PCD during cancer metastasis. Oral (*O*) SCC proliferation, migration, invasion, and metastasis are controlled by miR-218, a tumor-suppressing miRNA that is often downregulated [11]. Lai et al. found that the upregulation of miR-31-5p in OSCC resulted in significant increases in free fatty acid accumulation, lipid droplet production, and OSCC cell migration and proliferation [12]. Although several studies have investigated the roles of miRNAs in the onset and progression of HNC, none has investigated the potential of necroptosis-related miRNAs in predicting the prognosis of patients with HNC. Therefore, further research on molecular markers for predicting the prognosis of patients with HNC utilizing necroptosis-related miRNA is needed.

In this study, we first collected miRNA expression data and related clinical data from patients with HNC from The Cancer Genome Atlas (TCGA), and we extracted data for the miRNAs associated with necroptosis that were identified from the literature. Then, a prognostic multimiRNA molecular marker was developed based on differential expression analysis and uni- and multivariate Cox regression analyses. Finally, the target genes of miRNAs associated with prognosis were identified and subjected to functional enrichment analysis to evaluate the potential mechanisms of the prognostic miRNAs.

## 2. Materials and Methods

### 2.1. Search Strategy, Data Collection, Preprocessing, Normalization, and Integrated Analysis

The UCSC Xena Browser (https://xenabrowser.net/datapages/) was used to obtain miRNA expression data (log_2_ (FPKM+1)) (fragments per kilobase per million) of patients with HNC and related clinical information from TCGA. The dataset information is shown in Table 1. Patients for whom survival, new event, or survival status was uncertain were deleted. miRNA expression data were also obtained from TCGA (https://tcgadata.nci.nih.gov/tcga/findArchives.htm). Next, we collected the following 13 cancer metastasis-related necroptosis miRNAs from the literature [10]: miR-495, miR-331-3p, miR-15a, miR-148a-3p, miR-7-5p, miR-141-3p, miR-425-5p, miR-200a-5p, miR-210, miR-223-3p, miR-500a-3p, miR-181-5p, and miR-16-5p. We extracted expression data of these necroptosis-related miRNAs for data matching, filtering, and correction, as well as filtering and matching with the relevant clinical data for subsequent analysis.

### 2.2. Establishment of a Prediction Model Based on Necroptosis-Associated miRNAs

The *R* software package limma [13] was used to assess differential expression miRNA expression between tumor and nontumor tissues in the processed data, using the following filtering criteria: |log_2_(fold change)| > 0 and false discovery rate < 0.05. Necroptosis-related miRNAs that satisfied these criteria were considered differentially expressed. The necroptosis-related miRNAs were subjected to univariate and batch univariate Cox regression analyses. Based on the results thereof, miRNAs were selected for multivariate Cox regression to develop a prognostic risk model. For the uni- and multivariate Cox regression analyses, the model's risk score was integrated with clinical variables. Based on the findings of these analyses, we assessed whether the risk score may be used as an independent predictor. The model's prediction capacity was assessed by constructing a receiver operating characteristic (ROC) curve and calculating the area under the curve (AUC). Patients were divided into low-risk and high-risk groups using the median risk score value as a cut-off, and a risk-related survival curve was created.

### 2.3. Identification and Analysis of Necroptosis-Associated miRNA Target Genes

The target genes of necroptosis-associated miRNAs that were strongly associated with prognosis were predicted from the miRDB, TargetScan, and miRTarBase databases. Target genes that were identified in all three databases were selected as the target genes of the necroptosis-related miRNAs. We used the Cytoscape software to construct a miRNA–target gene network and the *R* package clusterProfiler to analyze Gene Ontology (GO) and the Kyoto Encyclopedia of Genes and Genes (KEGG) term enrichment of the target genes [14].

## 3. Results

### 3.1. Prognostic Necroptosis-Associated miRNA Model

**Figure 1:**
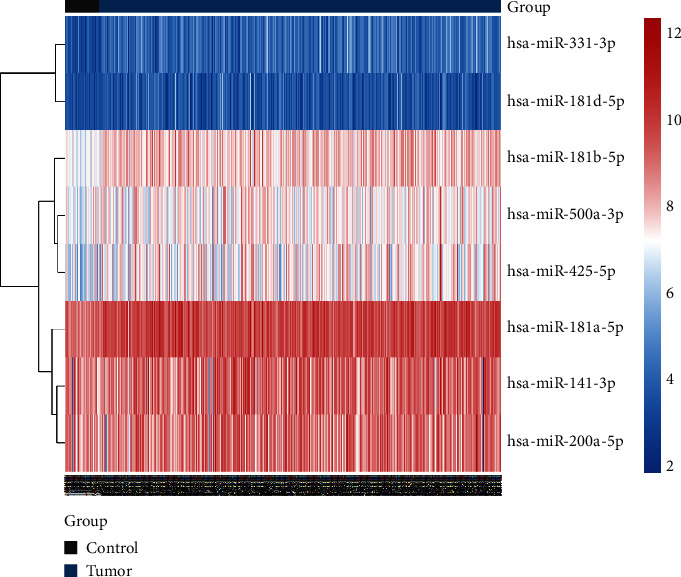
Heatmap of eight differently expressed miRNAs between head and neck cancer and normal tissues. Red and blue represent up- and downregulated expression in tumor, respectively.

**Figure 2:**
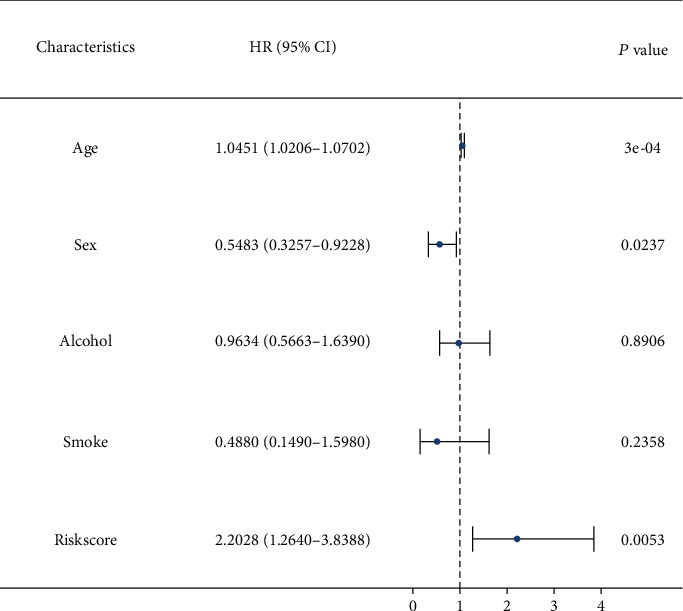
Forest plot for univariate Cox regression analysis.

**Figure 3:**
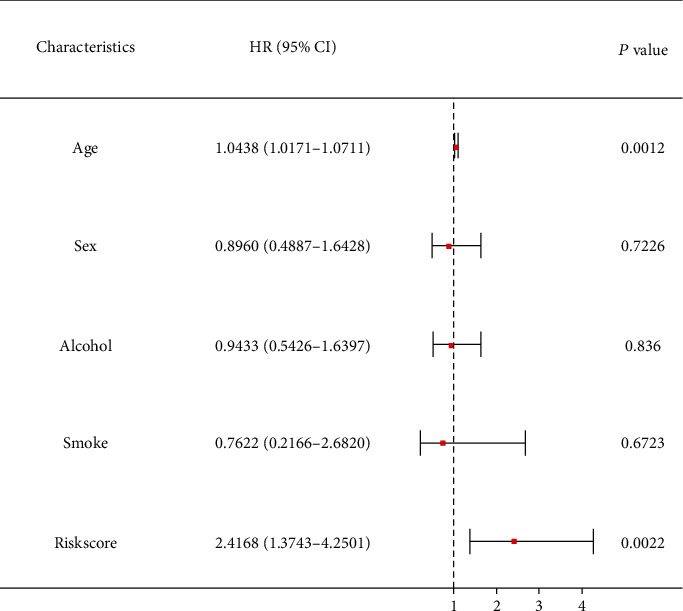
Forest plot for multivariate Cox regression analysis.

**Figure 4:**
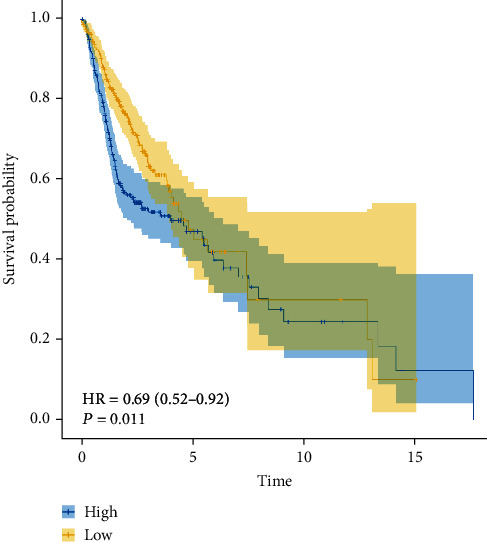
Survival curves for high- and low-risk score groups of patients with head and neck cancer.

**Figure 5:**
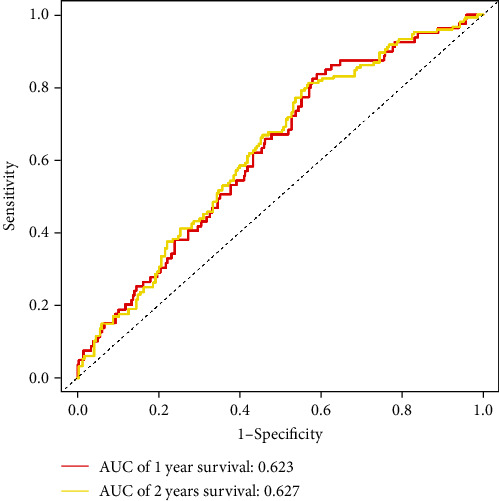
Time-dependent ROC curves for 1- and 2-year survival.

Eight miRNAs that were overexpressed in HNC tissues as compared to normal tissues were identified through differential expression analysis: hsa-miR-331p, hsa-miR-181d-5p, has-miR-181b-5p, has-miR-500a-3p, hsa-miR-425-5p, hsa-miR-181a-5p, hsa-miR-141-3p, and hsa-miR-200a-5p (Figure 1). We selected these eight miRNAs to generate a risk model based on multivariate Cox regression and integrated the risk score with clinical variables for uni- and multivariate Cox regression analyses. The results indicated that the risk score had potential as an independent prognostic factor. The risk score increased with poorer prognosis (univariate Cox regression results: hazard ratio (HR): 2.2028, 95% confidence interval (CI): 1.2640–3.8388, *P* = 0.0053, Figure 2; multivariate Cox regression results: HR: 2.4168, 95 percent CI: 1.3743–4.2501, *P* = 0.0022, Figure 3). Survival curve analysis revealed that patients with a high-risk score had a dismal HNC prognosis (*P* = 0.0109, Figure 4). ROC curve analysis indicated that the model has a certain predictive capacity (Figure 5).

### 3.2. Results of miRNA Target Gene Enrichment Analysis

**Figure 6:**
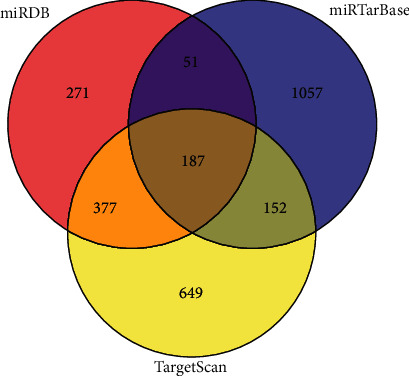
Target genes of miRNAs identified in the TargetScan, miRDB, and miRTarBase public databases.

**Figure 7:**
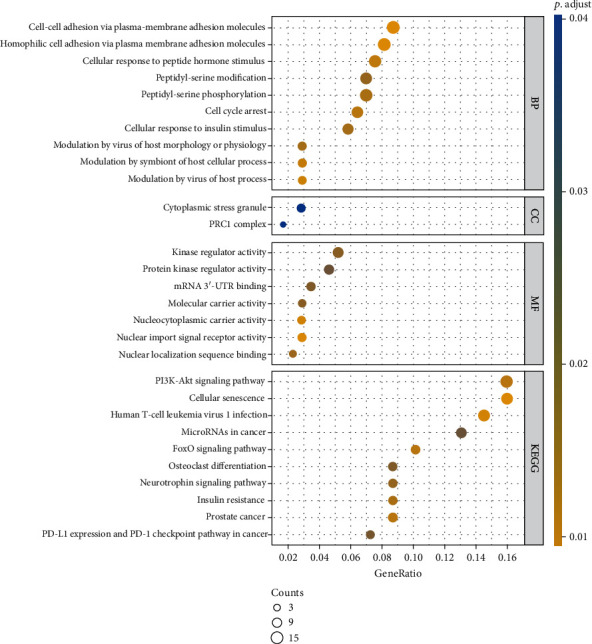
Gene Ontology (GO) analysis of the target genes of has-miR-181b-5p.

We selected has-miR-181b-5p, which was the most differentially expressed in HNC, for target gene prediction, and we obtained 187 target genes (Figure 6). The genes were subjected to GO and KEGG enrichment analyses. The genes were significantly (*P* < 0.05) enriched in the following GO terms: biological process: “homophilic cell adhesion via plasma membrane adhesion molecules,” “cell-cell adhesion via plasma-membrane adhesion molecules,” “modulation by virus of host process,” “modulation by symbiont of host cellular process,” and “cellular response to peptide hormone stimulus;” cellular component: “PRC1 complex” and “cytoplasmic stress granule;” and molecular function: “nuclear import signal receptor activity,” “nucleocytoplasmic carrier activity,” “nuclear localization sequence binding,” “molecular carrier activity,” kinase regulator activity,” “mRNA 3′-UTR binding,” and “protein kinase regulator activity.” KEGG enrichment analysis showed that the target genes were mostly related to “cellular senescence,” “human T-cell leukemia virus 1 infection,” “FoxO signaling pathway,” “prostate cancer,” and “PI3K-Akt signaling pathway” (Figure 7). These findings indicated that has-miR-181b-5p likely is related to these functions and pathways.

## 4. Discussion

As one of the most widespread malignancies in the world, HNC is particularly prevalent in underdeveloped nations [15]. Although there have been significant advances in HNC diagnosis and treatment methods and concepts in recent years, there are still problems that urgently need to be solved. Accordingly, new therapeutic targets and drugs and their molecular mechanisms for the treatment of HNC and the prevention of HNC recurrence are major HNC research topics.

Cancer cells often move from the original site through lymphatics, blood arteries, and body cavities for local invasion and then spread throughout the body and colonize new locations [16]. In recent years, ncRNAs have been shown to affect the transition of epithelial mesenchymal cells and tumor angiogenesis in cancer metastasis. Small nuclear RNAs, miRNAs, short interfering RNAs, PIWI-interacting RNAs, small nucleolar RNAs, circular RNAs, and lncRNAs are some of the most common types of ncRNAs. Genome expression during tumor growth and metastasis is controlled by various mechanisms. NcRNAs have been revealed to play a role in controlling cell death during cancer spreads.

To our knowledge, this was the first study to investigate the potential of necroptosis-related miRNAs in HNC prognostication. We first identified 13 necroptosis-related miRNAs associated with cancer metastasis in the literature, eight of which (has-miR-331-3p, has-miR-181d-5p, has-miR-181b-5p, has-miR-500a-3p, hsa-miR-425-5p, hsa-miR-181a-5p, has-miR-141-3p, and has-miR-200a-5p) were used to establish a risk model based on their differential expression.

SNHG20 interacts with has-miR-331-3p to activate Her2 in tumors, enhancing tumor cell invasion and migration [17]. RIP1 plays a critical role in the occurrence of necroptosis upon complexation with RIP3 [18]. In animals and cells, high concentrations of hsa-miR-200a-5p promote RIP3-induced necroptosis [19]. Cell death occurs as a result of RIP3 activating MLKL, which then moves to the cell membrane to alter membrane permeability and then translocates back to the nucleus for protein degradation. TargetScan analysis revealed that hsa-miR-500a-3p has MLKL-biding sites. MLKL-mediated necroptosis was considerably reduced in HK2 human tubular epithelial cells treated with cisplatin treatment, which reduced hsa-miR-500a-3p production [20]. Atrazine induced necroptosis in carp lymphocytes by inhibiting miR-181-5p, thus activating the immune system and boosting glycolysis [21]. In intestinal endothelial cells, hsa-miR-141-3p suppressed lipopolysaccharide-induced necroptosis through its effect on RIPK1 [22]. In septic liver damage, hsa-miR-425-5p has been shown to suppress necroptosis by interacting with RIP1 and directly lowering the RIP1 level, thus lowering the inflammatory response and acute liver damage [23].

This study had some limitations. Firstly, we built the model entirely based on data from the public database TCGA, and we did not enroll participants to obtain clinical data for model validation. Secondly, we did not validate the expression, function, and mode of action of the miRNAs included in the predictive model.

## 5. Conclusion

Our study showed that necroptosis is closely related to HNC, as several necroptosis-related miRNAs were significantly differentially expressed between cancerous and noncancerous head and neck tissues. Our model, including eight necroptosis-related miRNAs, can be used to predict HNC prognosis. The new miRNA markers for HNC prognosis identified in this study provide a foundation for the discovery of additional necroptosis-related genes in the future.

## Figures and Tables

**Table 1:** Characteristics of dataset in this study.

Public dataset	Sample size		Platform
	Controls	Patients	
TCGA head and neck cancer (HNSC)	44	485	IlluminaHiSeq_miRNASeq

## Data Availability

The data used to support the findings of this study are included within the article.

## Abbreviations

ncRNA: Noncoding RNA
miRNA: MicroRNA
RIP1: Receptor-interacting protein kinase 1
RIP3: Receptor-interacting protein kinase 3
GO: Gene Ontology
KEGG: Kyoto Encyclopedia of Genes and Genomes.
